# Supplementary material for: Urinary C3 levels associated with sepsis and acute kidney injury—A pilot study
Source: PLoS One. 2021 Nov 12;16(11):e0259777. doi: 10.1371/journal.pone.0259777 (PMC8589214; doi:10.1371/journal.pone.0259777)

Fig 1A. C3a/C3 Dot Blot. Urine samples obtained at consecutive time points from ICU patients suffering from serious infections. Patient (**o**) did not survive this episode due to multiorgan failure the same day as urine sample #5 was collected. Shown is a representative experiment out of four. Left panel of the figure represents patients with decreasing urinary C3a/C3 levels. Right panel shows patients with increasing urinary C3a/C3 levels towards 5th and 6th day of ICU treatment.

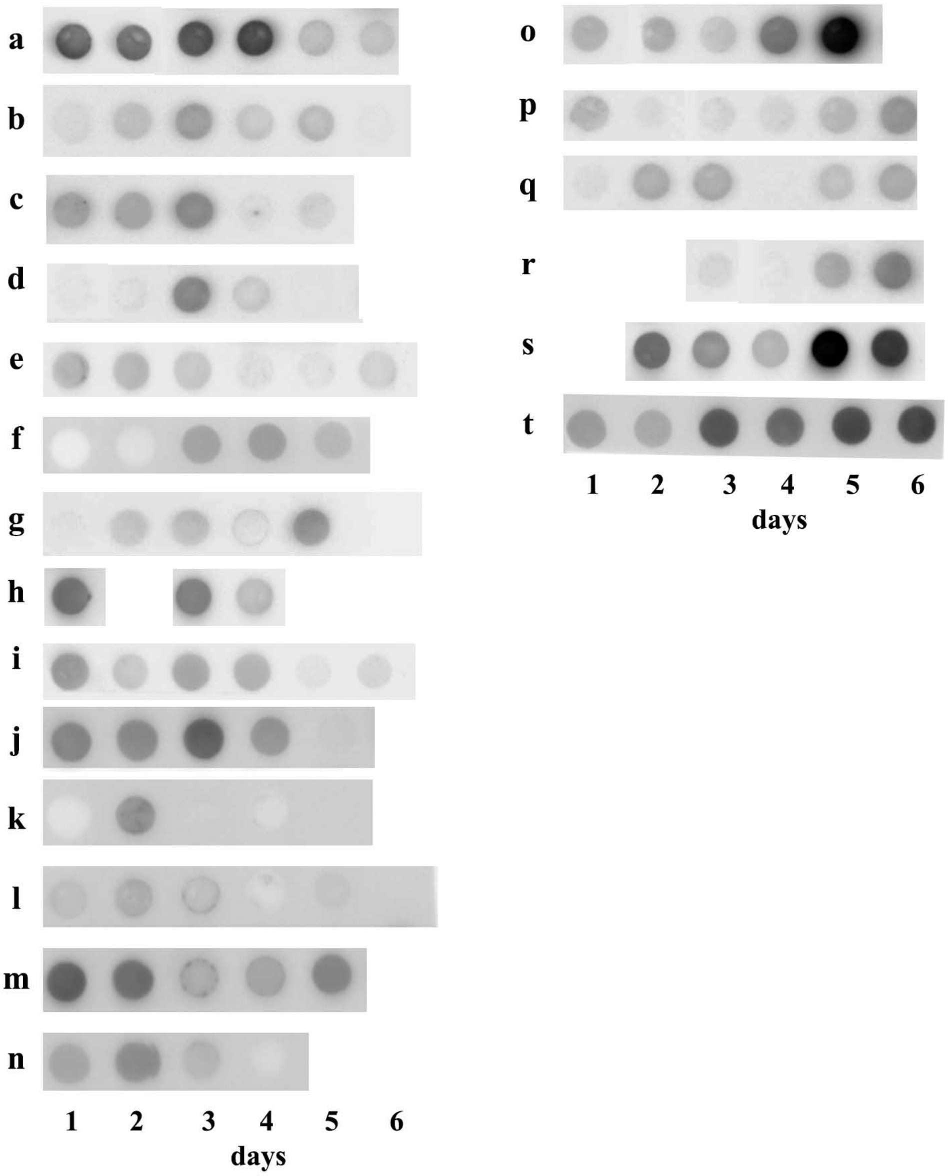

This represents the original strips of the individual patients in a day-to-day sequence. Urine was not available from each patient at each time point, therefore these time points were left blank.

Original out of which Fig 1B was assembled

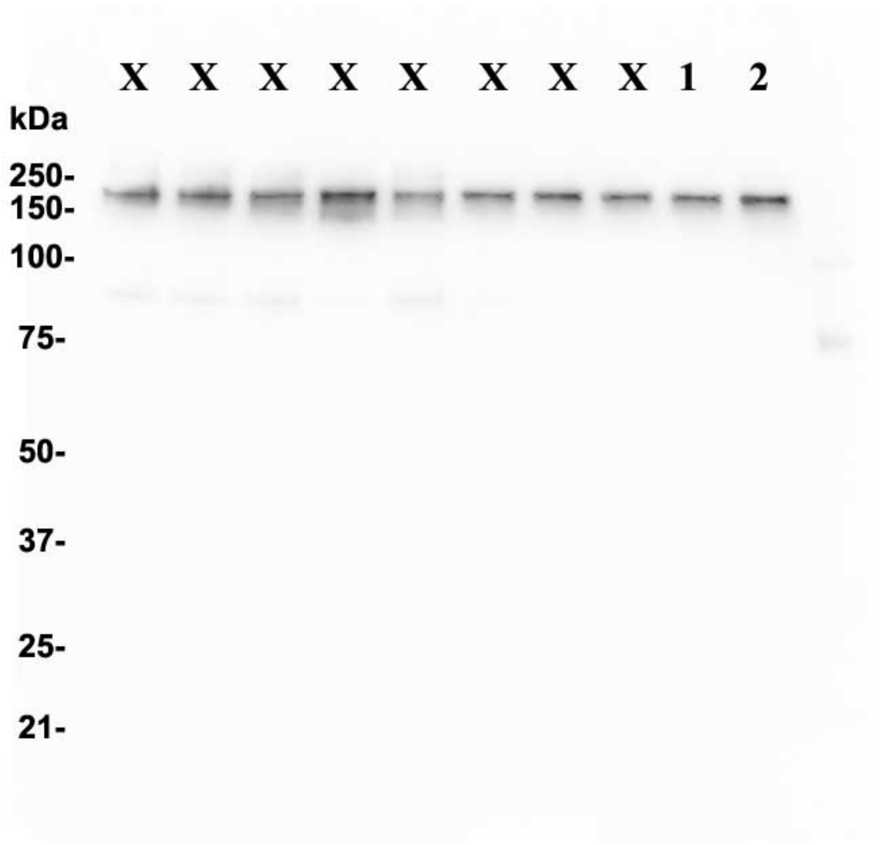

Blot containing serum samples

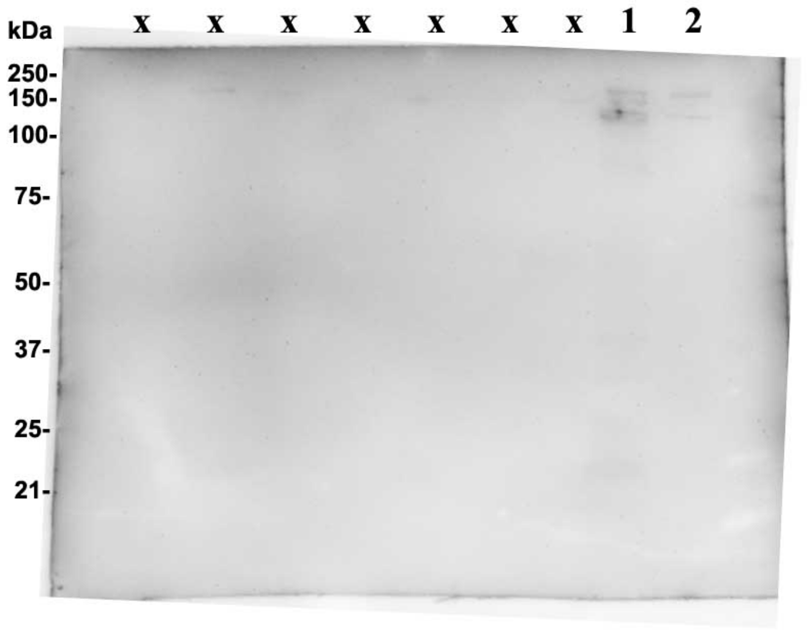

Blot containing urine samples

Original blot for Fig 1C

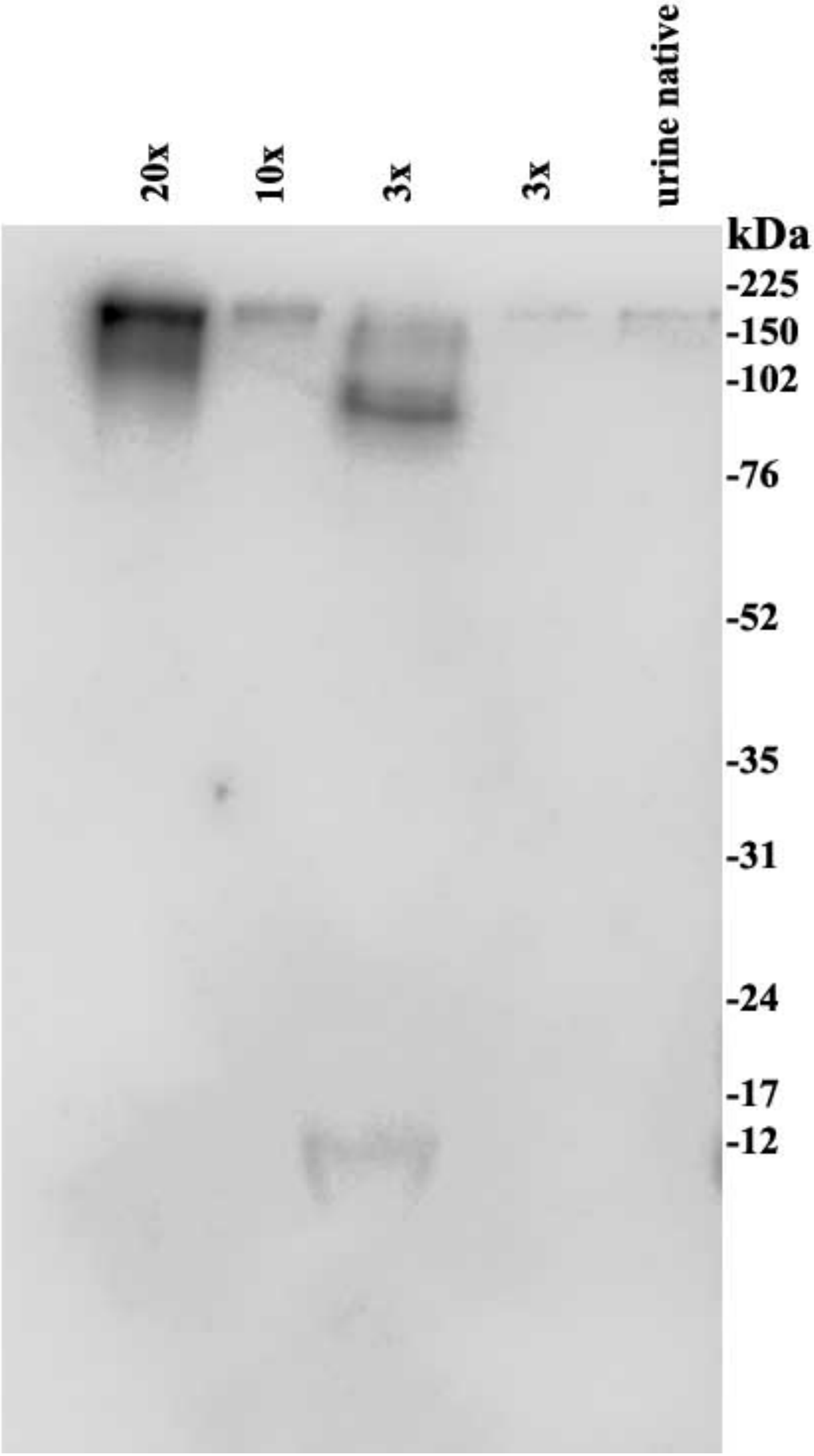

Supplement: S1 Raw images — (PDF) [file pone.0259777.s001.pdf]
