# Supplementary material for: Urinary C3 levels associated with sepsis and acute kidney injury—A pilot study
Source: PLoS One. 2021 Nov 12;16(11):e0259777. doi: 10.1371/journal.pone.0259777 (PMC8589214; doi:10.1371/journal.pone.0259777)
Supplement: Attachment — Submitted filename: point-to-point response PlosONEdocx.docx [file pone.0259777.s002.docx]

Daniela Gerges, MD, MSc

Division of Nephrology and Dialysis

Department of Medicine III,

Medical University of Vienna,

Waehringer Guertel 18-20,

1090 Vienna, Austria

[daniela.gerges@meduniwien.ac.at](mailto:daniela.gerges@meduniwien.ac.at)

______________________________________

Editors in Chief

**PLOS ONE**

______________________________________

Vienna, July 6^th^, 2021

**Resubmission - “Are urinary C3 levels associated with the renal acute phase reaction in acute kidney disease? – A pilot study” (PONE-D-21-05396R1)**

Dear Editors in Chief,

After submitting an extensive revision and point-to-point response concerning our manuscript “**PONE-D-21-05396R1 Are urinary C3 levels associated with the renal acute phase reaction in acute kidney disease? – A pilot study**”, the article was rejected. However, we considered that we could comprehensively address reviewers’ concerns. Therefore, we present here a rebuttal letter including our point-by-point response to each of the comments in our decision letter.

Please find enclosed our point-to-point response to each of the comments made in our decision letter for the manuscript entitled **Are urinary C3 levels associated with the renal acute phase reaction in acute kidney disease? – A pilot study (PONE-D-21-05396R1)***.*

We kindly ask you, to reconsider publication in your esteemed journal.

Yours sincerely,

Daniela Gerges, MD, MSc

(former Daniela Knafl)

Reviewer comments: black font

Response: blue font

Modifications in the revised version of the manuscript: red font

Major concerns:

**Reviewer #1:**

In light of the critics of the reviewer we would be inclined to change the title of the manuscript towards: **Urinary C3 levels associated with sepsis and acute kidney injury – A pilot study.**

(C1) The authors cannot trace the source of urine C3. Even though other studies and the authors found an expression of C3 from kidney cells, it is still very likely that most of the C3 found in the urine is from the liver. The authors proposed that the C3 is too high in molecular weight so that it cannot pass the filtration barrier. However, immunoglobulins are frequently found to pass through the same barrier in kidney conditions and they have similar molecular weight. These claims are thus not convincing.

(R1A): We hereby attempt to defend and clarify our experience and position, although we understand and have included the reviewer's statements as strong arguments: As mentioned before, local synthesis of C3 has already been shown in the past by Sacks S et al. Sack S et al described the capability of glomerular mesangial cells to produce C3 and C4, with an increase in C4 expression after stimulation with interferon-gamma, whereas C3 expression remains unaffected under interferon-gamma stimulation [1]. These data are substantiated by a further study, which demonstrates that C3 is synthesized, processed, and secreted by glomerular epithelial cells under basal conditions, with the C3 alpha and beta polypeptide chains having identical electrophoretic mobilities with those of hepatic C3. In contrast to the study mentioned above, stimulation with interferon-gamma lead to an increase in C3 gene expression, indicating that C3 expression in glomerular epithelial cells is regulated by interferon-gamma [2]. In a further study using human samples of patients with postischemic acute renal failure, local synthesis of C3 in the tubule was described and the authors were able to distinguish tubular C3 from C3 of hepatic origin [3]. In addition, another study described enhanced local C3 production in immune-mediated nephritis [4].

IgG is ascribed a size of 150 kDa, whereas C3 is estimated to be significantly larger at 190 kDa; moreover, the molecular structure of IgG is quite different from that of C3. This argues against the reviewer's hypothesis that IgG excretion can be taken as a comparative molecule to C3 in its amount of excretion through the slit diaphragm of the glomerulus in sepsis.

In addition, in all individuals included in the Human Protein Atlas, which is open access, C3 is expressed to variable extent such as shown below. The extracted figure below represents the individual CAB004209, Female, age 56 and was stained for C3 by a rabbit monospecific antibody.

**Reviewer material I.** This figure has not been produced by our research group. It represents one extract from the human protein atlas: <https://www.proteinatlas.org/ENSG00000125730-C3/tissue/kidney#img>. Further images can be seen obtained each from another individual even in children age 7, but all express C3.

Concerning our study, immunofluorescence staining clearly demonstrates tubular epithelial cells containing intracellular C3 (Fig. 7 in the manuscript).

Figure 7 from main manuscript

**Fig 7. Immunofluorescence staining of cryosections of a kidney removed because of a renal cell carcinoma using rabbit anti human C3-specific antibody.** Tubular epithelial cells contain intracellular C3 (left panel). Merging C3 staining with DAPI nuclear staining (right panel).

Two modes of secretion are known in biology. It has to be assumed that C3 is not constitutively secreted into urine. There must be a regulated mode of secretion from nephronic epithelial cells. A specific stimulus secretion coupling has to exist. One of them might occur in infection and sepsis. The exact triggering molecule is not characterized but it must differ from that in the liver.

To further support and confirm our data, we performed C3-specific RT-qPCR transcriptome analysis from urine sediments of AKI patients with and without kidney transplantation.

This study was initiated in response to reviewer comments. These data were performed for review purposes only and were not included in our manuscript because they provide data from a partially different cohort. This is because urine sediment was only available from a small number of study participants. In this analysis, forty-six samples showed no expression of C3 by cycle 44 and were therefore considered negative. Seventy-nine samples had C3 transcripts in the cells or cell fragments of the urine sediment, indicating C3 synthesis.

**Methods:** 125 urine samples of a total of 64 patients were utilized. Twenty-four patients were kidney transplant recipients, one patient had received bone marrow transplantation and all patients experienced AKI. Urine sediment was palleted and RNA was extracted out of the sediment by mixing with Trizol. The Trizol lysate was then mixed with chlorophorm for precipitating the total RNA using isopropanol, as described in the Trizol test manual. Purified RNA was dissolved in RNAse free water and mixed with dNTPs random hexamer primers and reverse transcriptase using superscript enzyme. The resulting cDNA was diluted 1:4 with H_2_O and amplified using C3 specific probes from TaqMan^®^ (Hs01100881_m1, Thermo Fisher Scientific) and 2x TaqMan^®^ Universal Master Mix in a StepOnePlus qPCR machine (Applied Biosystems^®^). Data recording was performed over 46 cycles. Individual C3 expression levels in terms of cycle threshold (Ct) were normalized using GAPDH as house-keeping gene resulting in a ΔCt value, and further calculated using the ΔΔCt method [5].

**Results:** Forty-six samples showed no expression of C3 until 44 cycles and were therefore considered negative. Seventy-nine samples exhibited C3 transcripts in urine sediment cells or cell fragments, indicative for C3 synthesis. Relative C3 expression normalized to GAPDH is given below in **Reviewer material II**.

**Reviewer material II. Relative C3 expression in urinary sediments of AKI patients normalized to GAPDH.** 125 urine samples of 64 patients were analyzed: 24 patients were kidney transplant recipients; one patient had received bone marrow transplantation and all patients experienced AKI. Each bar represents one patient. Values are given as relative expression of C3 normalized to GAPDH in urinary sediments.

Furthermore, we have again included a further analysis only performed for the purpose of review, which shall serve to validate our data. It exhibits that also in contrast nephropathy excretion of C3/C3a in urine is increased one day after administration of contrast agent. Therefore, it might be assumed that C3/C3a is upregulated or the secretion out of the tubular cells is stimulated in response to the adverse effect caused by the contrast agent or might exhibit cell-protective effects, such as autophagy. At this point it must be mentioned that none of these patients exhibited signs of infection and did not show elevated CRP levels. These data are nevertheless content of another study and are shown here for the sole purpose of review to again substantiate our data and will not be included in the present study but are elucidated for the reviewer.

**Reviewer material III. Urinary C3/C3a levels increase one day after intravenous CT contrast agent application.** 45 patients were included in this work. Day 1 represents urinary C3/C3a levels prior to contrast agent application. Urinary C3/C3a levels increased on day 2, after application of contrast-CT and went down on day 3. Before contrast application all patients were checked for being negative for any inflammatory process.

What seems very probable is that C3 is synthesized locally in tubular cells, as has been described in prior literature. We have included a statement about the origin of C3 with the available literature to our discussion section within the previous revision.

Page 13, paragraph 1, Discussion,

Local synthesis of C3 has already been described in the past. Sack S and colleagues were able to describe that glomerular mesangial cells are capable of producing C3 and C4, with an increase in C4 expression after stimulation with INF𝛾, whereas C3 expression remains unaffected under interferon-gamma stimulation [1]. These data are substantiated by a further study, which demonstrates that C3 is synthesized, processed, and secreted by glomerular epithelial cells under basal conditions, with the C3 alpha and beta polypeptide chains having identical electrophoretic mobilities with those of hepatic C3. In contrast to the study mentioned above, stimulation with INF𝛾 lead to an increase in C3 gene expression, indicating that C3 expression in glomerular epithelial cells is regulated by INF𝛾 [2]. In addition, increased local C3 synthesis has been described in human diseases, such as postischemic acute renal failure and immune-mediated nephritis [3, 4].

An according section has been added to the discussion section.

Discussion page 14-15:

Two modes of secretion are known in biology. It has to be assumed that C3 is not constitutively secreted into urine, but that there must be a regulated mode of secretion from nephronic epithelial cells. A specific stimulus secretion coupling appears to exist with one of them occurring in infection and sepsis. The exact triggering molecule has not been characterized yet but must differ from that in the liver. Transcription factor and repressor proteins are usually expressed in a tissue- and cell subtype-specific manner. Thereby, renal epithelial cells express different transcription factors and repressor proteins than hepatocytes. The function of these proteins is to interact with specific responsive elements on the DNA of genes. In recent years, miRNAs have been characterized as similar players at the mRNA level and thereby can regulate translation and mRNA turnover. As a result, different tissues are able to respond differently to the same stimulus such as interleukins, toxic compounds, drugs, and nutrients. This response is achieved by regulating gene expression and protein production. With regard to our study, we must hypothesize that the C3 gene in the kidney is regulated differently than the CRP gene in the liver. Moreover, the dynamics of CRP in serum is much brisker than that of C3.

At this point the question should be elaborated, why urinary C3a/C3 levels correlate negatively with serum CRP (p<0.0001) in our study. The consumption and activation of C3 is a well-known marker for determining the activity of inflammatory diseases, such as rheumatic diseases or atypical hemolytic uremic syndrome. In this context, a decrease in C3 represents increased inflammation. Consequently, C3 can be said to represent an inverse acute phase parameter. In relation to our study, it is important to mention that mAb 3F7E2-based ELISA detects both C3 and C3a. To clarify the activation of C3 at the site of renal secretion, a commercially available C3a ELISA was performed that showed the presence of C3a in urine on all 6 days of measurement. The hypothesis that this measured C3a originates from the blood, because it is a small molecule and, unlike C3, could pass through the glomerular filter, must be rejected because C3a has a very short half-life and is rapidly degraded in the blood by proteases.

Overall, the question arises as to the purpose of this urinary C3 provided by the kidney.

(C2) The authors did not exclude the possibility that the C3 production and the kidney damage are both independently correlated to the sepsis, while C3 production/leakage and kidney damage are not linked in the current study.

(R2) Transcription factor and repressor proteins are expressed in a tissue- and cell subtype-specific manner. For example, renal epithelial cells express different transcription factors and repressor proteins than hepatocytes. The function of these proteins is to interact with specific responsive elements on the DNA of genes. In recent years, miRNAs have been characterized as similar players at the mRNA level and thereby can regulate translation and mRNA turnover. As a result, different tissues are able to respond differently to the same stimulus such as interleukins, toxic compounds, drugs, and nutrients. This response is achieved by regulating gene expression and protein production. With regard to our study, we must hypothesize that the C3 gene in the kidney is regulated differently than the CRP gene in the liver. Moreover, the dynamics of CRP in serum is much more brisk than that of C3.

(C3) The authors claimed that the urinary C3 an “indicator for an acute phase reaction and cellular stress condition to the kidneys”. However, according to the experimental method, the authors did not trace the course of disease to define an acute phase. Refining an acute phase of sepsis is very difficult to achieve in practice. Data show that C3 and CRP concentration are negatively correlated, which is the opposite of the conclusion, as CRP is one of the best indicators of acute phase reaction. The authors did not examine cellular stress of the kidney either. These conclusions are not supported by the data.

(R3): As described in the Methods section under the subheading “sample collection” only patients admitted to the intensive care unit (ICU) fulfilling at least 4 of 10 sepsis criteria were included into this study [6]. The sepsis criteria were established to define an acute phase of sepsis and are therefore the adequate tool to substantiate the fact that our patients were acutely ill. It is true, that CRP and urinary C3a/C3 levels correlated negatively with serum CRP (p<0.0001), however the mAb 3F7E2 based ELISA detects both, C3 and C3a. Consumption and activation of C3 is a known marker for determining the activity of inflammatory diseases, such as rheumatic diseases or atypical hemolytic uremic syndrome. Thereby, a decrease of C3 stands for increased inflammation. Therefore, it can be said, that for example like serum albumin, C3 represents an inverse acute phase parameter.

It is true that the CRP level is the best indicator of an acute-phase response, which is orchestrated by the liver and represents the response to a pathological stimulus. However, we disagree with the reviewer's assumption that the CRP level may be one of the best indicators of an acute-phase response in the kidney. The site in the kidney directly affected by sepsis is the endothelium and signaling to the tubular epithelial cells and podocytes occurs with a time delay. The tubular epithelial cells undergo specific response processes involving IFN signaling and STAT1. The kidney has different modalities to respond, often delayed, such as upregulation of alpha-2-macroglobulin, while CRP is not found elevated or not elevated in the blood in such disease [7].

An attempt to follow the reviewer's suggestion to better represent the acute phase reaction in the kidney, we wanted to elucidate the activation of C3 at the site of renal secretion, a commercially available C3a ELISA was performed in response to an acute phase reaction, and the data have already been included in the previous review. Again, the reviewer might say that this is from blood, since C3a is a small molecule. This is contradicted by the assumption that C3a has a very short half-life and is rapidly degraded by proteases in the blood.

Methods, page 7, paragraph 2

**Urine C3a ELISA**

The human C3a ELISA (Invitrogen, BMS2089) was carried out as indicated in the test manual. Urine samples were thawed under airflow. The diluted (1:4 or 1:2 in sample diluent) urine samples (100µl) were pipetted into the prewashed test wells and incubated for two hours under constant shaking together with the provided standard series which was prepared in the sample diluent. Following washing on an automated ELISA washer (three times) the biotin-conjugate prepared in the assay buffer (100µl) was pipetted into each test well and incubated for one hour at RT under constant shaking. Following a second washing step again three times, the streptavidin-HRP prepared in the assay buffer provided with the test kit (100µl) was added into each well and incubated for one hour under constant shaking at RT. After a final washing step, the TMB substrate solution (100µl) was pipetted into each well and reacted at RT for 20 minutes under light protection. The reaction was then stopped by adding 100µl stop solution. The test signal was read at 450nm at an ELISA reader and the sample concentrations were calculated according to the standard curve using the Gen5 version 2.03 program.

Results, page 9-10, paragraph 3

As depicted in the left panel of **Fig 2** the highest mean overall level of C3a/C3 was found on the second day of measurement, which represented the second day of treatment (all p<0.05). Selectively measured C3a was detectable in urine over all 6 days (**Fig 2** right panel). Out of 230 samples 178 exhibited detectable levels of C3a as shown in (**Fig 2** right panel).

**Fig 2. ELISA-measurement of urinary complement factor C3a/C3 and C3a in 85 ICU patients.** Data are presented in columned scatter graphs; the horizontal lines mark the mean values of the respective C3a/C3 (left) and C3a (right) levels. Day 1: n=78; day 2: n=78; day 3: n=72; day 4: n=66; day 5: n=56; day 6: n=48.

**References**

1.	Sacks S, Zhou W, Campbell RD, Martin J. C3 and C4 gene expression and interferon-gamma-mediated regulation in human glomerular mesangial cells. Clin Exp Immunol. 1993;93(3):411-7. Epub 1993/09/01. PubMed PMID: 8370168; PubMed Central PMCID: PMC1554924.

2.	Sacks SH, Zhou W, Pani A, Campbell RD, Martin J. Complement C3 gene expression and regulation in human glomerular epithelial cells. Immunology. 1993;79(3):348-54. Epub 1993/07/01. PubMed PMID: 8406564; PubMed Central PMCID: PMC1421987.

3.	Farrar CA, Zhou W, Lin T, Sacks SH. Local extravascular pool of C3 is a determinant of postischemic acute renal failure. Faseb J. 2006;20(2):217-26. Epub 2006/02/02. doi: 10.1096/fj.05-4747com. PubMed PMID: 16449793.

4.	Sacks SH, Zhou W, Andrews PA, Hartley B. Endogenous complement C3 synthesis in immune complex nephritis. Lancet. 1993;342(8882):1273-4. Epub 1993/11/20. PubMed PMID: 7901586.

5.	Livak KJ, Schmittgen TD. Analysis of relative gene expression data using real-time quantitative PCR and the 2(-Delta Delta C(T)) Method. Methods. 2001;25(4):402-8. Epub 2002/02/16. doi: 10.1006/meth.2001.1262. PubMed PMID: 11846609.

6.	Funk D, Sebat F, Kumar A. A systems approach to the early recognition and rapid administration of best practice therapy in sepsis and septic shock. Curr Opin Crit Care. 2009;15(4):301-7. Epub 2009/06/30. doi: 10.1097/MCC.0b013e32832e3825. PubMed PMID: 19561493.

7.	Menon R, Otto EA, Hoover P, Eddy S, Mariani L, Godfrey B, et al. Single cell transcriptomics identifies focal segmental glomerulosclerosis remission endothelial biomarker. JCI Insight. 2020;5(6). Epub 2020/02/29. doi: 10.1172/jci.insight.133267. PubMed PMID: 32107344; PubMed Central PMCID: PMCPMC7213795.
